# Targeting Endothelial Cell Metabolism by Inhibition of Pyruvate Dehydrogenase Kinase and Glutaminase-1

**DOI:** 10.3390/jcm9103308

**Published:** 2020-10-15

**Authors:** Céline A. Schoonjans, Barbara Mathieu, Nicolas Joudiou, Luca X. Zampieri, Davide Brusa, Pierre Sonveaux, Olivier Feron, Bernard Gallez

**Affiliations:** 1 Biomedical Magnetic Resonance Research Group, Louvain Drug Research Institute, Université Catholique de Louvain (Uclouvain), 1200 Brussels, Belgium; celine.schoonjans@uclouvain.be (C.A.S.); barbara.mathieu@student.uclouvain.be (B.M.); 2 Pole of Pharmacology and Therapeutics, Institut de Recherche Expérimentale et Clinique, Université Catholique de Louvain (Uclouvain), 1200 Brussels, Belgium; luca.zampieri@uclouvain.be (L.X.Z.); pierre.sonveaux@uclouvain.be (P.S.); olivier.feron@uclouvain.be (O.F.); 3 Nuclear and Electron Spin Technologies, Louvain Drug Research Institute, Université Catholique de Louvain (Uclouvain),1200 Brussels, Belgium; nicolas.joudiou@uclouvain.be; 4 CytoFlux-Flow Cytometry Platform, Institut de Recherche Expérimentale et Clinique, Université Catholique de Louvain (Uclouvain), 1200 Brussels, Belgium; davide.brusa@uclouvain.be

**Keywords:** endothelial cells metabolism, tumor microenvironment, glycolysis inhibition, dichloroacetate, glutaminolysis inhibition, BPTES, ^13^C-NMR

## Abstract

Targeting endothelial cell (EC) metabolism should impair angiogenesis, regardless of how many angiogenic signals are present. The dependency of proliferating ECs on glucose and glutamine for energy and biomass production opens new opportunities for anti-angiogenic therapy in cancer. The aim of the present study was to investigate the role of pyruvate dehydrogenase kinase (PDK) inhibition with dichloroacetate (DCA), alone or in combination with the glutaminase-1 (GLS-1) inhibitor, Bis-2-(5-phenylacetamido-1,3,4-thiadiazol-2-yl) ethyl sulfide (BPTES), on Human umbilical vein endothelial cells (HUVECs) metabolism, proliferation, apoptosis, migration, and vessel formation. We demonstrated that both drugs normalize HUVECs metabolism by decreasing glycolysis for DCA and by reducing glutamate production for BPTES. DCA and BPTES reduced HUVECs proliferation and migration but have no impact on tube formation. While DCA increased HUVECs respiration, BPTES decreased it. Using both drugs in combination further reduced HUVECs proliferation while normalizing respiration and apoptosis induction. Overall, we demonstrated that DCA, a metabolic drug under study to target cancer cells metabolism, also affects tumor angiogenesis. Combining DCA and BPTES may reduce adverse effect of each drug alone and favor tumor angiogenesis normalization.

## 1. Introduction

Once activated by pro-angiogenic factors, endothelial cells (ECs) sprout to extend the vasculature and to supply the growing tumors with oxygen and nutrients [1]. While angiogenesis is an enticing target, therapies based on vascular endothelial growth factor (VEGF) blockade have shown limited results due to upregulation of alternative proangiogenic growth factors [1,2]. In contrast, targeting EC metabolism should impair angiogenesis, regardless of how many angiogenic signals are present [1] The dependency of proliferating ECs on glucose but also glutamine and fatty acids for energy and biomass production opens new opportunities for anti-angiogenic therapy in cancer [3,4,5]. High rate of glycolysis is a hallmark of proliferating ECs, allowing a rapid ATP production and the generation of precursors for biomass synthesis [4,6]. Even if glycolysis is crucial for ECs proliferation, mitochondria in ECs are still functional and can be fueled by several metabolites such as glutamine [1]. It has been shown that glutamine, the most abundant free amino acid, is essential for EC proliferation and migration [5,7]. Glutamine is metabolized into glutamate through glutaminase-1 (GLS-1), which can fuel the mitochondrial tricarboxylic acid (TCA) cycle [5,7,8]. Together with TCA cycle intermediates, glutamine and glutamate are involved in antioxidant defense production, synthesis of nucleotides, proteins, and fatty acids. Consequently, counteracting glycolysis and glutamine pathways are promising approaches to reduce angiogenesis. In this respect, it has been previously shown that blockade of the glycolytic activator PFKFB3 reduced cancer cell invasion, intravasation, and metastasis by normalizing tumor vessels [6]. It has also been shown that the inhibition of GLS-1 impaired ECs survival, proliferation, and migration [5,7,8,9].

Based on the above considerations, we can hypothesize that inhibition of pyruvate dehydrogenase kinase (PDK) could decrease proliferative ECs metabolism and impact ECs migration and proliferation. The rationale behind this hypothesis is the following: PDK phosphorylates and inactivates the enzyme pyruvate dehydrogenase (PDH). The active unphosphorylated PDH metabolizes pyruvate to acetyl-CoA that fuels the TCA cycle. Therefore, PDK inhibition reactivates PDH, leading to a redirection of glucose metabolism from cytoplasmic glycolysis to mitochondrial oxidation. It is already known that PDK plays a role in angiogenesis through the hypoxia-inducible factor-1α (HIF-1α). In 2017, a study on flow-induced metabolic reprogramming in ECs reported that HIF-1α increased PDK-1, which reduced mitochondrial respiratory capacity [10]. On cancer cells from various origins, it has been shown that DCA, a PDK inhibitor currently studied in clinical trials, decreased HIF-1 activity, resulting in a reduced expression of HIF-1α target genes including pro-angiogenic factors [11,12] and a decreased tumor angiogenesis [12,13,14,15,16]. To our knowledge, besides this HIF-1α mediated effect by cancer cells, a direct effect of DCA through a metabolic reprogramming of ECs has not been previously considered. This is in sharp contrast with the established metabolic reprogramming induced by DCA on cancer cells [17]. By reversing aerobic glycolysis in cancer cells, DCA reduces glycolytic intermediates available for proliferation, mainly through the pentose phosphate pathway [12]. Consequently, this decrease in building blocks from glycolysis is responsible for the anti-proliferative effects of PDK inhibition observed in various cancer cells lines [12,17,18,19,20,21]. In addition, by reactivating the mitochondrial activity, DCA reverses the abolition of mitochondria-dependent apoptosis [12,22]. Of note, it was recently observed in cancer cells that DCA exposure may also exacerbate glutamine metabolism providing the rationale for the combination of DCA with the GLS-1 inhibitor, bis-2-(5-phenylacetamido-1,3,4-thiadiazol-2-yl) ethyl sulfide (BPTES) [12].

As glycolysis and glutamine pathways are essential for ECs fitness, the aim of the present study was to investigate the role of PDK inhibition with DCA, alone or in combination with the GLS-1 inhibitor, BPTES, on HUVECs metabolism, proliferation, apoptosis, migration, and vessels formation.

## 2. Experimental Section

### 2.1. Cell Culture and Reagents

Primary Umbilical Vein Endothelial Cells (HUVECs) (ATCC^®^ PCS-100-013™, ATCC, Manassas, VA, USA), SiHa cells, and HCT-116 cells were acquired from the American Type Culture Collection (ATCC, Manassas, VA, USA). SiHa and HCT-116 cell lines were maintained in DMEM (D5030, Sigma-Aldrich, Saint-Louis, MO, USA) supplemented with 10 mM of glucose, 2 mM of glutamine, 10% heat-inactivated FBS (Thermo Fisher Scientific, Waltham, MA, USA) and with 25 mmol/L of both PIPES and HEPES. HUVECs were used until passage 5 and grown in Vascular Cell Basal Medium (ATCC^®^ PCS-100-030™, ATCC, Manassas, VA, USA) supplements with Endothelial Cell Growth Kit-VEGF (ATCC^®^ PCS-100-041™, ATCC, Manassas, VA, USA). Endothelial Cell Growth Kit-VEGF contains components that were added to Vascular Cell Basal Medium to create a complete, low serum culture environment. For each component, the final concentration in complete endothelial growth medium is as follows:

- rh VEGF: 5 ng/mL
- rh EGF: 5 ng/mL
- rh FGF basic: 5 ng/mL
- rh IGF-1: 15 ng/mL
- l-glutamine: 10 mM
- Heparin sulfate: 0.75 Units/mL
- Hydrocortisone: 1 µg/mL
- Ascorbic acid: 50 µg/mL
- Fetal bovine serum: 2%

Dichloroacetate (DCA) was ordered from Sigma-Aldrich (Saint-Louis, MO, USA) and dissolved in culture media. Bis-2-(5-phenylacetamido-1,3,4-thiadiazol-2-yl) ethyl sulfide (BPTES) was ordered from Sigma-Aldrich and first dissolved in DMSO (Sigma-Aldrich, Saint-Louis, MO, USA), and then diluted in culture media (final DMSO concentration ≤ 0.1%).

### 2.2. Cell Proliferation

Cell proliferation was assayed with a 5-bromo-2′-deoxyuridine (BrdU)-ELISA based kit (Roche, Bâle, Switzerland) following the provider’s instructions. A total of 2000 cells per well were seeded in a 96-well plate. After 24 h, cells were treated with 5 or 10 mM DCA and/or 1 µM BPTES. After 48 h exposure, cells were incubated in the presence of BrdU for 2 h. The amount of BrdU incorporated in the cells was quantified by measuring the absorbance at 370 nm using a SpectraMax M2e plate reader (Molecular Devices, San Jose, CA, USA, États-Unis), which permitted the quantification of DNA synthesis in proliferative cells.

### 2.3. Cell Migration

A total of 8000 cells per well were seeded in a culture-insert 2 well (μ-Dish35mm, Ibidi, Munich, Germany). After 24 h, 1 µg/mL MitomycinC (MitoC, Sigma-Aldrich) was added in each well to stop cellular proliferation. After 10 h, the insert was removed, creating a cell-free gap of 500 μm. The dish was filled with 2 mL of media containing 1 µg/mL MitoC and 5 or 10 mM of DCA and/or 1 µM BPTES. Pictures of migration were taken at T0 and T0 + 14 h with an inverted microscope (Life Cell Observer Z1 with AxioCam 504 mono). Cell migration was quantified with the wound healing tool ImageJ analysis software.

### 2.4. Tube Formation

24-well plates were coated with 300 µL per well of Matrigel growth factor reduced (Corning, NY, USA). Once the Matrigel had solidified, 30,000 cells were seeded and treated with 5 or 10 mM of DCA and/or 1 µM BPTES. After 4 h incubation, pictures were taken with an inverted microscope (Life Cell Observer Z1 with AxioCam 504 mono). Tube formation was quantified using WimTube of Wimasis image analysis system (Wimasis GmbH, Munich, Germany).

### 2.5. Western Blotting Analyses of PDH Phosphorylation

Whole cellular lysates were collected after 24 h of incubation with 5 or 10 mM DCA and immunoblot analysis were performed as previously described [23]. The following primary antibodies were used: phospho-PDHE1-A (1/1000, #ABS204, Millipore, Burlington, MA, USA) and PDHE1a (1:1000, #3820, Cell Signaling, Danvers, MA, USA). HSP90 antibody (1/1000, 610419, BD Bioscience, San Jose, CA, USA) was used for gel loading normalization. Densitometry analysis was performed using Image J software. Band densities for PDHE1α and *p*-PDHE1α were normalized to the band density of HSP90 in the same sample. Results are expressed in percentage of the control condition.

### 2.6. ^13^C NMR Spectroscopy Quantification of Metabolites

Isotope-labeled glucose medium was prepared with DMEM media (D5030, Sigma-Aldrich) supplemented with Endothelial Cell Growth Kit-VEGF and with 5 mmol/L of d-Glucose-^13^C_6_ (Sigma-Aldrich). Isotope-labeled glutamine medium was prepared with vascular Cell Basal Medium supplemented with Endothelial Cell Growth Kit-VEGF except the glutamine component. Instead of the glutamine component, 10 mM of l-glutamine-5-^13^C (Sigma-Aldrich) was supplemented in the isotope-labeled glutamine medium. A total of 48 h after seeding 4 × 10^5^ cells in a 100 mm dish (2 dishes per condition), cell culture medium was changed for isotope-labeled glucose or glutamine medium. Cells were incubated in the presence of 5 or 10 mM DCA and/or 1 µM BPTES from ^13^C-glutamine experiments. A total of 24 h after treatments, intra and extracellular metabolites were extracted as previously described [20]. For each condition, extra dishes were seeded, and protein quantification was performed using the BCA Protein Assay Kit (Pierce™ BCA Protein Assay Kit, Thermo Fisher). Results were quantified regarding the ratio of proteins levels between control and each treated condition.

### 2.7. Oxygen Consumption Rate

The effect of DCA and BPTES on the oxygen consumption rate was measured by Seahorse XF96 bioenergetic analyzer using the XF cell mito stress kit (Agilent Technologies), which allows to calculate basal OCR, maximal OCR after FCCP stimulation (carbonyl cyanide 4-(trifluoromethoxy)phenyl-hydrazone), and non-mitochondrial respiration after rotenone rotenone/antimycin A injection. In total, 2000 cells per well were seeded on XF96 culture plates. After 24 h, cells were treated with 5 or 10 mM DCA and/or 1 µM BPTES. After 24 h exposure, culture media were replaced by DMEM containing 5 mM glucose, 10 mM glutamine. Cells were incubated for 1 h in a CO_2_-free incubator before analysis. To obtain basal and maximal mitochondrial OCR, non-mitochondrial OCR was subtracted to basal and maximal OCR. Data were normalized to cell numbers quantified right before OCR measurement using a SpectraMax miniMax 300 imaging cytometer.

### 2.8. Flow Cytometry Measurement of Apoptosis

A total of 1 × 10^5^ cells were seeded in a 60 mm dish. After 24 h, cells were incubated with in the presence of 5 or 10 mM DCA and/or 1 µM BPTES. A total of 48 h after treatment, cells were rinsed with PBS, trypsinized, and resuspended in fresh media. Following provider’s instructions, EBioscience™ Annexin V Apoptosis Detection Kit APC (Invitrogen™, Thermo Fisher Scientific) was used for the measurement of dead, apoptotic, and living cells. The cells were distinguished between dead: late apoptotic (AnnV+DAPI+), early/medium apoptotic (AnnV+DAPI−), and alive (double negative). Flow cytometry data were collected using a BD FACS Canto II flow cytometer and analyzed with FlowJo software. FACS analysis by FlowJo were processed for each cell line separately. At least 20,000 events were analyzed for each sample.

### 2.9. Statistical Analysis

Data are expressed as mean ± SEM (standard error of the mean). The number of replicates and the statistical test used are indicated in each figure caption. GraphPad Prism 8.0 software (GraphPad Software, San Diego, CA, USA) was used to analyze all the data.

## 3. Results

### 3.1. DCA Reduces HUVECs Proliferation Similarly to Cancer Cells and Induces a Metabolic Shift from Glycolysis to Mitochondrial Respiration through PDH Activation

**Figure 1:**
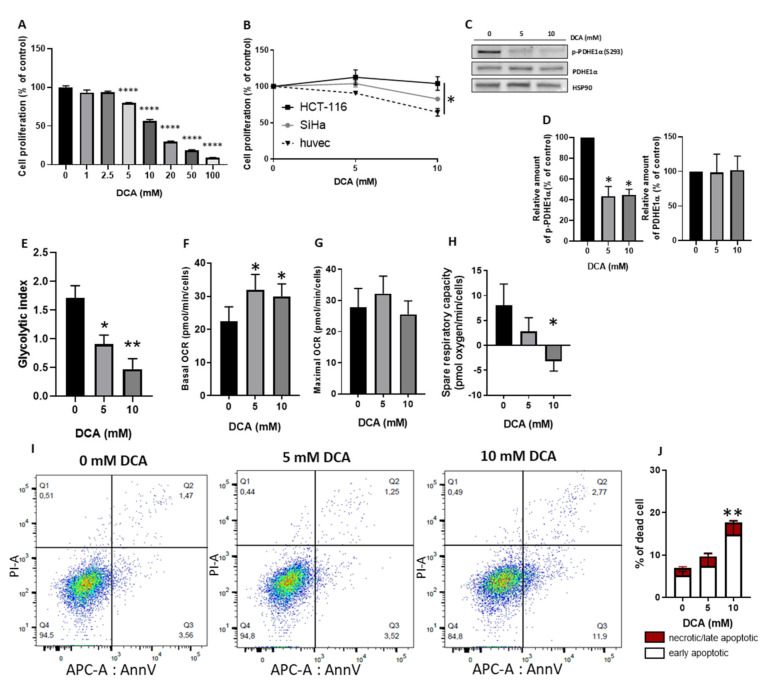
DCA reduces HUVECs proliferation and, through PDH activation, induces a metabolic shift towards decrease in glycolysis and in spare respiratory capacity. (**A**) proliferation of HUVECs exposed to increasing doses of DCA (0–100 mM) (*n* = 4). (**B**) comparison of the effect of DCA exposure on cell proliferation between cancer cells (HCT-116 and SiHa) and HUVECs (*n* = 3). (**C**) representative immunoblotting of total (PDHE1α) and phosphorylated (*p*-PDHE1α) form of PDH in HUVECs after exposure to 0, 5 or 10 mM DCA (*n* = 3). (**D**) densitometry analysis of *p*-PDHE1α and PDHE1α. (**E**) lactate production from glucose in control and DCA treated HUVECs (5 and 10 mM DCA); results are presented as ratio of total lactate (sum of intra-and extracellular lactate) on glucose uptake (*n* = 3). (**F**,**G**), oxygen consumption rate (**F**, basal OCR and **G**, maximum OCR) measured in HUVECs treated with or without DCA (5 and 10 mM) (*n* = 3). (**H**) spare respiratory capacity (maximal OCR minus basal OCR). (**I**) representative AnnexinV/PI flow cytometry plots of HUVECs treated with or without 5 or 10 mM of DCA. (**J**) histograms of apoptotic and dead cells (*n* = 4). Statistical analysis: One-way (**A**,**D**–**H**), or Two-way ANOVA (**J**) (Sidak’s multiple comparison test), * *p* < 0.05, ** *p* < 0.01, **** *p* < 0.0001.

We first investigated if HUVECs were sensitive to DCA. We observed that the exposure to increasing concentration of DCA was correlated with a reduction in HUVECs proliferation (Figure 1A). We determined that DCA at 5 mM was the lowest DCA concentration that induces a significant decrease in HUVECs proliferation. We compared our results with DCA effect on cancer proliferation (Figure 1B). We used two cancer cell lines: HCT-116 (colorectal cancer cells) and SiHa (cervix cancer cells).

We noticed that HUVECs were as sensitive to DCA exposure concentration than the SiHa cancer cell line and more sensitive than the HCT-116 cancer cell line. We then measured that these effects observed on HUVECs proliferation were associated to PDK inhibition, i.e., to a reduction in phosphorylated PDH. In HUVECs exposed to 5 or 10 mM DCA, a reduction in phosphorylated PDH was observed (Figure 1C,D). Next, we explored the consequences of PDK inhibition on HUVECs glycolytic metabolism. Using ^13^C-NMR spectroscopy, we followed the metabolism of glucose. As previously observed by others [4], we observed that proliferative HUVECs present a high glycolytic metabolism with a glycolytic index of 1.71 (ratio of lactate production on glucose consumed) (Figure 1E). The HUVECs exposure to 5 or 10 mM DCA led to a reduction in the glycolytic index (Figure 1E). To explore the impact of DCA on the mitochondrial activity of HUVECs, we measured the mitochondrial oxygen consumption rate (OCR). We observed an increase in mitochondrial basal OCR after DCA exposure (Figure 1F). However, HUVECs exposure to high concentration of DCA (10 mM) gives the same increase in OCR than exposure to the lower DCA concentration (5 mM). Using FCCP, we measured the maximal mitochondrial respiration rate and observed no difference with or without DCA exposure (Figure 1G). We then calculated the spare respiratory capacity (maximal OCR minus basal OCR) and noticed a reduction in spare respiratory capacity after DCA treatment (Figure 1H). Finally, we noticed that only the higher DCA concentration significantly increased HUVECs apoptosis (Figure 1I,J), while HUVECs exposure to 5 mM of DCA did not induce change in apoptosis.

### 3.2. DCA Reduces HUVECs Migration without Impacting Tube Formation

**Figure 2:**
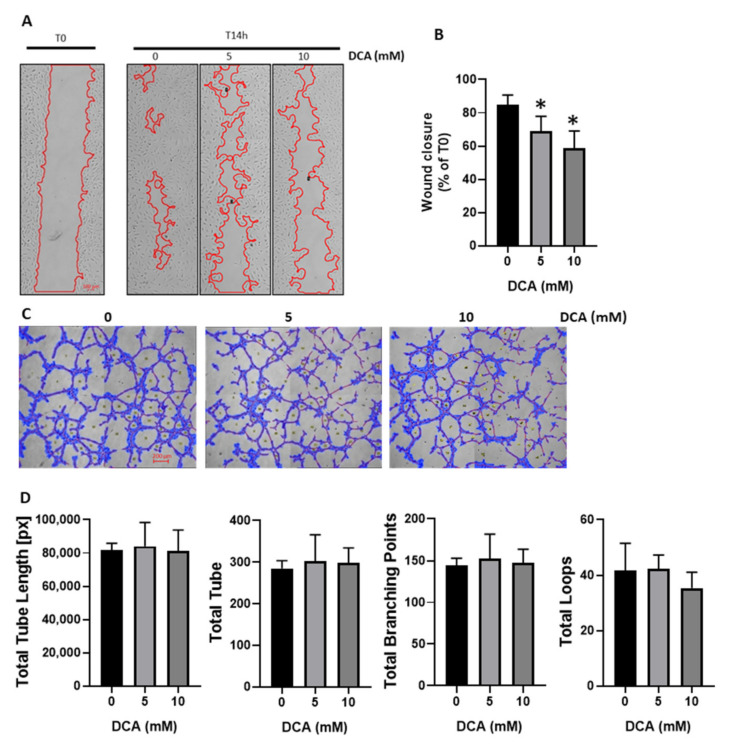
DCA reduces HUVECs migration without impact on tube formation. (**A**,**B**), Wound healing assays: HUVECs were pre-treated with mitomycin C and DCA treatment started when inserts were removed (T0), percentage of wound closure is calculated after 14 h (T14 h) of cell migration (*n* = 4). A, representative phase contrast microphotographs of wound healing assay. B, percentage of wound closure after 14 h calculated from each T0 counterparts. (**C**,**D**), Tube formation assays: HUVECs were seeded on Matrigel for 4 h with or without DCA treatment (*n* = 3). C, representative phase contrast microphotographs of tube formation assays. D, quantitative analysis of specific parameters (total tube length, total number of tubes, total branching point and total loops) of tube formation assays. Statistical analysis: One-way ANOVA (**B**,**D**) (Sidak’s multiple comparison test), * *p* < 0.05.

To explore the relevance of the DCA impact on HUVECs in vitro angiogenesis, we evaluated itseffect on HUVECs migration and tube formation (Figure 2). Using culture-inserts 2-well, we monitored the cell migration. To avoid the impact of cell proliferation, cells were incubated with mitomycin C, a cell division blocker. A total of 14 hours after removal of the insert, we observed that HUVECs migration was significantly lower in DCA treated cells compared to control cells (Figure 2A,B). We also measured HUVECs tube formation by seeding the cells on coated wells with Matrigel. A total of 4 h after cells seeding, tubes were formed. We quantified the total tube length, the number of tubes, the number of branching points (where three or more tubes converge), and the number of loops (areas enclosed by tubes) (Figure 2C,D). We measured no difference on tube formation between control and DCA treated HUVECs including all the quantified parameters.

### 3.3. Inhibition of Glutamine Conversion to Glutamate Reduces Huvecs Proliferation, Migration and Respiration without Change in Apoptosis Induction and Tube Formation

**Figure 3:**
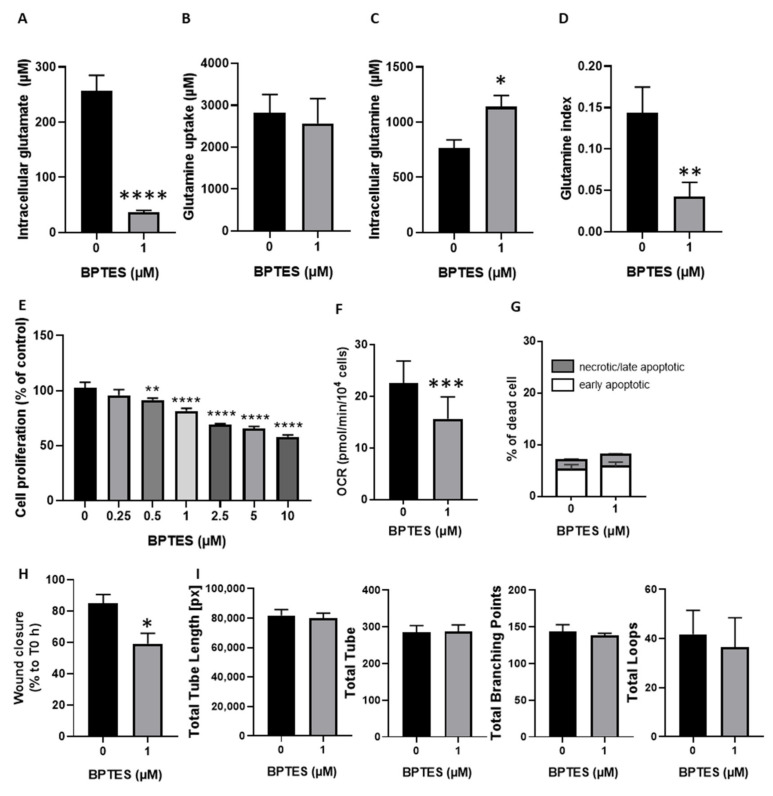
Inhibition of glutamine conversion to glutamate reduced HUVECs proliferation, migration, and respiration without change in apoptosis induction and tube formation. (**A**–**D**), HUVECs were exposed during 24 h to 10 mM of l-glutamine-5-^13^C and co-incubated with (or without) BPTES. Intra- and extracellular metabolites were quantified by ^13^C NMR spectroscopy (*n* = 5). (**A**), glutamate production from glutamine (intracellular concentration of glutamate) (**B**), glutamine uptake (extracellular concentration of glutamine at T0 minus extracellular concentration of glutamine after 24 h). (**C**), intracellular glutamine concentration. (**D**), glutamine index, (ratio of glutamate production on glutamine uptake). (**E**), proliferation of HUVECs (% of control) treated with increasing doses of BPTES (0–10 µM) (*n* = 4). (**F**), oxygen consumption rate (OCR), measured in HUVECs treated with or without 1 µM of BPTES (*n* = 3). (**G**) percentage of apoptotic and dead HUVECs treated with or without 1 µM of BPTES (*n* = 4). (**H**) percentage of wound closure after 14 h calculated from each T0 counterparts. (**I**) quantitative analysis of specific parameters (total tube length, total number of tubes, total branching point, and total loops) of HUVECs tube formation after 4 h incubation on Matrigel in the presence (or not) of 1 µM of BPTES. Statistical analysis: Paired *t*-test (**A**–**D**,**F**,**H**,**I**), One-way (**E**), or Two-way ANOVA (**G**) (Sidak’s multiple comparison test), comparison from control, * *p* < 0.05, ** *p* < 0.01, *** *p* < 0.001, **** *p* < 0.0001.

After glycolysis, glutaminolysis is the second most described metabolic pathway in ECs. We used the glutaminase-1 inhibitor, BPTES, to evaluate the impact of the inhibition of glutamine conversion to glutamate on HUVECs fitness. By ^13^C NMR, we first observed that BPTES reduces intracellular glutamate concentration by more than 80% (Figure 3A). This reduction did not affect the glutamine uptake (Figure 3B), but significantly increased intracellular glutamine concentration by more than 30% (Figure 3C). The glutamine index, which measures the production of glutamate from glutamine, was reduced by BPTES by more than 70% (Figure 3D). We then measured the impact of this inhibition on HUVECs proliferation. We observed that the exposure to increasing concentration of BPTES was correlated with a reduction in HUVECs proliferation (Figure 3E). We identified that 1 µM BPTES was the lowest concentration that significantly reduced HUVECs proliferation. We then evaluated if BPTES also induced changes in mitochondrial activity since glutamate is fueling the TCA cycle. We observed that BPTES significantly reduced the oxygen consumption rate (Figure 3F). We noticed that BPTES had no cytotoxic effect on HUVECs since we observed no significant change in apoptosis induction (Figure 3G). Finally, BPTES significantly reduced HUVECs migration, but, as observed for DCA, BPTES had no impact on tube formation (Figure 3H,I).

### 3.4. Combined Exposure to BPTES and DCA Increases the Impact on HUVECs Proliferation and Migration, but Normalize OCR and Apoptosis

**Figure 4:**
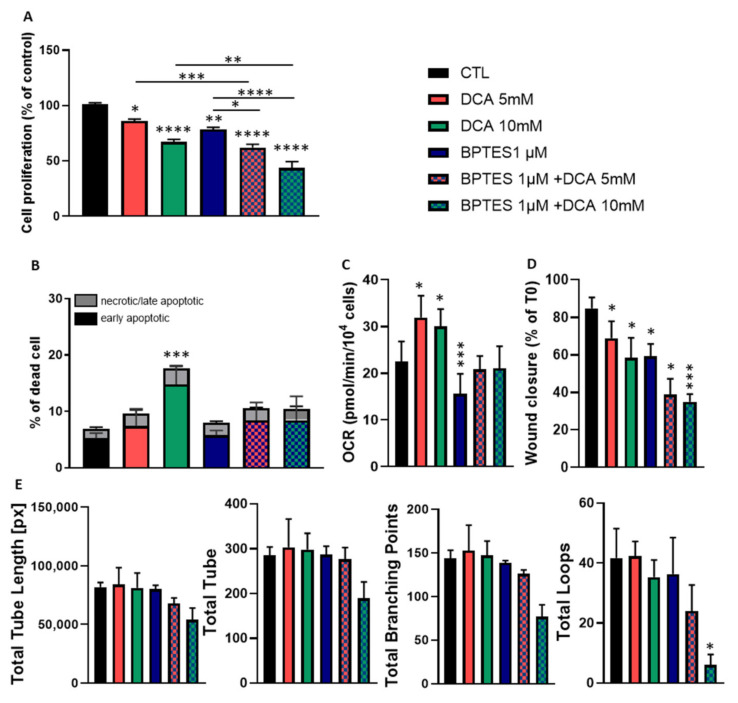
Combined exposure to DCA and BPTES enhances the reduction in HUVECs proliferation and migration but normalizes OCR and apoptosis. (**A**–**E**), HUVECs were treated with DCA (5 or 10 mM) and BPTES (1 µM), alone and in combination. The following parameters were evaluated: (**A**), cell proliferation (*n* = 4), (**B**), apoptotic and dead cells (*n* = 4), (**C**), oxygen consumption rate (*n* = 3), (**D**), wound closure (*n* = 3) and (**E**), tube formation (*n* = 3). Statistical analysis: One-way (**A**,**C**–**E**), or Two-way ANOVA (**B**) (Sidak’s multiple comparison test), comparison with control except for A where specific pairs are noted with a black line, * *p* < 0.05, ** *p* < 0.01, *** *p* < 0.001, **** *p* < 0.0001.

Since DCA and BPTES are targeting two different pathways that both regulate HUVECs metabolism, we investigated the impact of their combination. We first observed that the combined exposure to DCA (5 mM or 10 mM) and BPTES (1 µM) significantly decreased HUVECs proliferation, with a larger effect compared to the effect observed for each drug used alone (Figure 4A). Interestingly, the combination did not increase the cytotoxic effect when the DCA concentration was 5 mM. On the contrary, while DCA 10 mM significantly increased apoptosis when used alone, the combination of DCA 10 mM with BPTES had no significant impact on apoptosis (Figure 4B). We then measured the effect of the combination on OCR. We observed that the combination of both drugs abrogated the effects of either drug (Figure 4C). While DCA alone increased OCR, and BPTES alone decreased OCR, the combination of both drugs did not significantly change OCR compared to controls. Regarding HUVECs migration, we observed that the combined exposure to DCA and BPTES increased the reduction in migration induced by either drug used alone (Figure 4D). Finally, we measured the impact of the combination on tube formation. We observed that only the combination with the highest concentration of DCA (10 mM) impacted tube formation with a significant reduction in the number of branching points and the number of loops (Figure 4E).

### 3.5. BPTES and DCA Modulate Glutamine Metabolism and Reveal the Importance of Citrate on HUVECs Homeostasis

**Figure 5:**
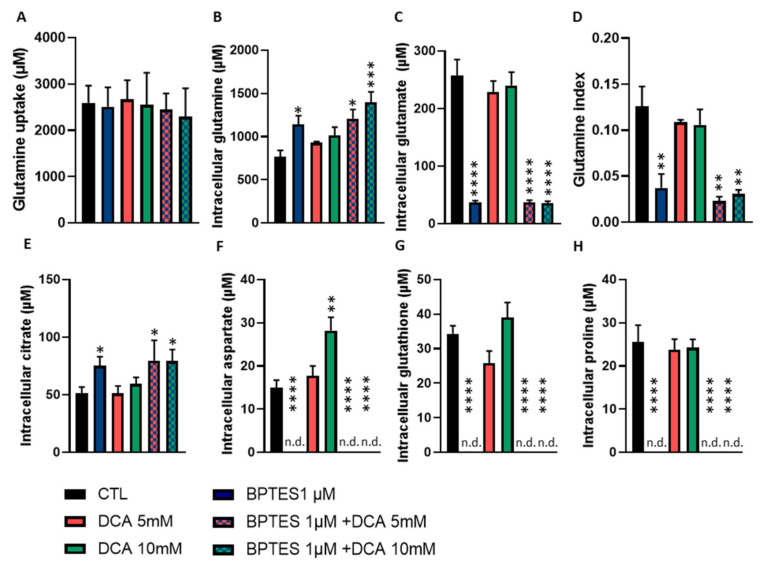
Downstream metabolites of glutamine are differentially impacted by BPTES and DCA. (**A**–**H**), HUVECs were exposed during 24 h to 10 mM of l-glutamine-5-13C and co-incubated with or without DCA (5 or 10 mM) and BPTES (1 µM), alone and in combination. Intra- and extracellular metabolites were quantified by ^13^C NMR spectroscopy (*n* = 5). (**A**), glutamine uptake. (**B**), intracellular glutamine concentration. (**C**), glutamate production from glutamine. (**D**), glutamine index, ratio of glutamate production on glutamine uptake. (**E**), citrate production from glutamine. (**F**), aspartate production from glutamine. (**G**), glutathione production from glutamine. (**H**), proline production from glutamine. Abbreviation: n.d., none detected. Statistical analysis: One-way ANOVA (**A**–**H**) (Sidak’s multiple comparison test), comparison from control, * *p* < 0.05, ** *p* < 0.01, *** *p* < 0.001, **** *p* < 0.0001.

To go further on the characterization of BPTES and DCA impact on endothelial cells metabolism, we monitored their impact on ^13^C-glutamine metabolism. We already observed that BPTES reduced the glutamate production and increased the intracellular glutamine concentration. We also observed that DCA alone had no impact on glutamine and glutamate concentration (Figure 5A–D). However, we noticed that DCA alone increased aspartate production from glutamine. We observed that DCA alone had no impact on glutathione, citrate, and proline production (Figure 5E–H). When HUVECs were exposed to BPTES, we observed no signal of aspartate, proline, and glutathione in the ^13^C-NMR spectra (Figure 5E–H). Interestingly, the signal of citrate was increased by BPTES, when used alone or in combination (Figure 5E).

## 4. Discussion

By demonstrating that HUVECs are sensitive to DCA (Figure 1A), we highlight two important facts. First, the use of DCA as anti-cancer treatment may affect cells in the tumor microenvironment other than cancer cells. Indeed, we observed that HUVECs are sensitive in the same range of DCA concentration than two different cancer cell lines (Figure 1B). The second important fact is that DCA may be considered as an alternative (or complementary) anti-angiogenic agent. Actually, we showed that DCA reduces HUVECs proliferation (Figure 1A) but only increases apoptosis at high DCA concentration (Figure 1I). This means that, depending on the dose, DCA may exhibit a cytostatic effect without inducing cytotoxicity, suggesting that there is room for a DCA dosage exhibiting antiangiogenic effects but sparing quiescent healthy ECs. This could be useful to normalize tumor angiogenesis, since tumor vasculature is functionally and structurally abnormal due to aberrant proangiogenic factors signaling leading to a hyper activation of ECs metabolism [1]. In order to reduce cancer cell invasion and to improve chemotherapy, there is a growing interest to normalize vessel formation in tumors through a remodeling of tumor endothelial cell metabolism [6]. As previously reported by others [6], we showed that HUVECs have a high glycolytic metabolism (Figure 1E), the majority of glucose entering the cell being metabolized into lactate. We also showed that, as in cancer cells [24], DCA reduces PDH phosphorylation in HUVECs, an observation consistent with the inhibition of PDK by DCA that allows pyruvate to fuel the TCA cycle (Figure 1C). Accordingly, we demonstrated that DCA was able to reduce this hyperglycolytic metabolism (reduction in the glycolytic index, Figure 1E) and to increase mitochondrial respiration (Figure 1F). By studying mitochondrial oxygen consumption, we showed that DCA stimulates OXPHOS activity in HUVECs. Moreover, we showed that DCA did not alter maximal respiratory capacity but decreased spare respiratory capacity. These data suggest that DCA did not increase the density of mitochondria. We observed that increase in OCR was the same at 5 and 10 mM DCA. We also observed that spare respiratory capacity was close to zero at a 5 mM DCA concentration, and all the respiratory capacity was used (negative value) at 10 mM DCA (Figure 1H). This may explain why we observed no difference between 5 and 10 mM DCA, respiration being at the maximum of capacity. Moreover, using the low DCA concentration (5 mM), we noticed that this increase in mitochondrial activity was not accompanied by an increase in apoptosis, apoptosis being induced only at the highest DCA concentration (Figure 1I,J). Finally, we observed that DCA reduced HUVECs migration (Figure 2B) without, however, impacting tube formation (Figure 2D). This discrepancy may arise from the nature of the assay matrices, Matrigel for tube formation assays and ibiTreat surface for migration assays, that may engage distinct integrins on HUVEC surface.

Altogether, our observations highlight that appropriate dosing of DCA is able to alter in vitro key steps in the angiogenic process, prompting for further investigations to evaluate to what extent this strategy could support vessel normalization.

**Figure 6:**
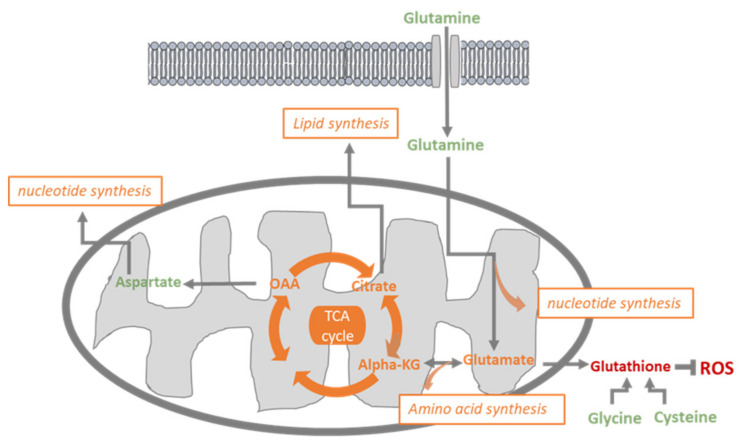
Glutamine metabolism: glutamine enters into the cell and can contribute to nucleotide synthesis or be converted into glutamate; glutamate participates in glutathione synthesis and can be converted to α-ketoglutarate (alpha-KG), which fuels the TCA cycle, which produces oxaloacetate (OAA) and citrate. OAA can be converted to aspartate and participate in nucleotide synthesis, citrate supports lipid synthesis (FAS).

As tumor angiogenesis is not only dependent on glycolysis but also on glutaminolysis [5,7], we investigated the impact of glutamine metabolism disruption on HUVECs. Using the glutaminase inhibitor, BPTES, we observed that inhibition of glutamate production from glutamine (Figure 3A) was associated with a reduction in HUVECs proliferation, respiration and migration (Figure 3E,F,H) but had no impact on apoptosis induction and tube formation (Figure 3G,I). Interestingly, although BPTES reduced glutamate production, citrate was still produced from glutamate (Figure 5A–E), even to a higher level than in control ECs. This observation suggests that the residual production of glutamate (less than 20%) in the presence of BPTES is used for citrate production possibly upon reductive carboxylation as previously described [7]. This underlines the crucial role of citrate for proliferating HUVECs metabolism in need of phospholipids to build up new lipid bilayers (Figure 6) [3]. Disruption of fatty acid synthesis was actually shown to reduce ECs proliferation and impair angiogenesis [3,25]. Of note, the deficit in glutamate availability is also very likely to influence non-mitochondrial pathways such as the glutathione and proline synthesis that we found to be completely inhibited in the presence of BPTES. More importantly, in BPTES-treated cells, we did not observe any NMR signal of aspartate (Figure 5F). This finding was at odds with the effect of DCA (in particular at 10 mM, Figure 5E) that increased aspartate production in HUVECs. These results can be related to the previously reported role of mitochondrial FA oxidation in aspartate production to support nucleotide synthesis in ECs [3]. We can therefore propose a model where DCA promotes the activity of the TCA cycle, and thereby supports aspartate production from oxaloacetate while glutamine acts as an essential anaplerotic substrate (so that blocking glutaminase has dramatic consequences on TCA cycling). PDK and glutaminase inhibition deserve to be studied in vivo in order to have reliable data about their impact on tumor angiogenesis. To measure the potential effects on tumor growth and metastasis, such an approach would first require conducting mouse experiments to select the optimal drug concentrations to normalize endothelial cell metabolism.

This study raises new opportunities for combining drugs acting on the metabolism, not only to target cancer cells directly, but also to target HUVECs. We showed that the metabolic reprogramming induced by both PDK and glutaminase inhibitors could affect tumor angiogenesis. However, it should be noted that, in this study, the impact of these drugs was evaluated on HUVECs, and it would be interesting to use other ECs models, such as human microvascular endothelial cells, to further validate our observations. Our findings warrant further research regarding a potential normalization of tumor angiogenesis with these metabolic inhibitors.

## References

[1] Li X., Sun X., Carmeliet P. (2019). Hallmarks of Endothelial Cell Metabolism in Health and Disease. Cell Metab..

[2] Fitzgerald G., Soro-Arnaiz I., De Bock K. (2018). The Warburg Effect in Endothelial Cells and its Potential as an Anti-angiogenic Target in Cancer. Front Cell Dev. Biol..

[3] Schoors S., Bruning U., Missiaen R., Queiroz K.C., Borgers G., Elia I., Zecchin A., Cantelmo A.R., Christen S., Goveia J. (2015). Fatty acid carbon is essential for dNTP synthesis in endothelial cells. Nature.

[4] De Bock K., Georgiadou M., Schoors S., Kuchnio A., Wong B.W., Cantelmo A.R., Quaegebeur A., Ghesquiere B., Cauwenberghs S., Eelen G. (2013). Role of PFKFB3-driven glycolysis in vessel sprouting. Cell.

[5] Huang H., Vandekeere S., Kalucka J., Bierhansl L., Zecchin A., Bruning U., Visnagri A., Yuldasheva N., Goveia J., Cruys B. (2017). Role of glutamine and interlinked asparagine metabolism in vessel formation. EMBO J..

[6] Cantelmo A.R., Conradi L.C., Brajic A., Goveia J., Kalucka J., Pircher A., Chaturvedi P., Hol J., Thienpont B., Teuwen L.A. (2016). Inhibition of the Glycolytic Activator PFKFB3 in Endothelium Induces Tumor Vessel Normalization, Impairs Metastasis, and Improves Chemotherapy. Cancer Cell.

[7] Kim B., Li J., Jang C., Arany Z. (2017). Glutamine fuels proliferation but not migration of endothelial cells. EMBO J..

[8] Peyton K.J., Liu X.M., Yu Y., Yates B., Behnammanesh G., Durante W. (2018). Glutaminase-1 stimulates the proliferation, migration, and survival of human endothelial cells. Biochem. Pharmacol..

[9] Eelen G., Dubois C., Cantelmo A.R., Goveia J., Bruning U., DeRan M., Jarugumilli G., Van Rijssel J., Saladino G., Comitani F. (2018). Role of glutamine synthetase in angiogenesis beyond glutamine synthesis. Nature.

[10] Wu D., Huang R.T., Hamanaka R.B., Krause M., Oh M.J., Kuo C.H., Nigdelioglu R., Meliton A.Y., Witt L., Dai G. (2017). HIF-1alpha is required for disturbed flow-induced metabolic reprogramming in human and porcine vascular endothelium. Elife.

[11] Sonveaux P., Copetti T., De Saedeleer C.J., Vegran F., Verrax J., Kennedy K.M., Moon E.J., Dhup S., Danhier P., Frerart F. (2012). Targeting the lactate transporter MCT1 in endothelial cells inhibits lactate-induced HIF-1 activation and tumor angiogenesis. PLoS ONE.

[12] Sutendra G., Michelakis E.D. (2013). Pyruvate dehydrogenase kinase as a novel therapeutic target in oncology. Front. Oncol..

[13] Sutendra G., Dromparis P., Kinnaird A., Stenson T.H., Haromy A., Parker J.M., McMurtry M.S., Michelakis E.D. (2013). Mitochondrial activation by inhibition of PDKII suppresses HIF1a signaling and angiogenesis in cancer. Oncogene.

[14] Duan Y., Zhao X., Ren W., Wang X., Yu K.F., Li D., Zhang X., Zhang Q. (2013). Antitumor activity of dichloroacetate on C6 glioma cell: In vitro and in vivo evaluation. Onco. Targets Ther..

[15] Michelakis E.D., Sutendra G., Dromparis P., Webster L., Haromy A., Niven E., Maguire C., Gammer T.L., Mackey J.R., Fulton D. (2010). Metabolic modulation of glioblastoma with dichloroacetate. Sci. Transl. Med..

[16] Kinnaird A., Dromparis P., Saleme B., Gurtu V., Watson K., Paulin R., Zervopoulos S., Stenson T., Sutendra G., Pink D.B. (2016). Metabolic Modulation of Clear-cell Renal Cell Carcinoma with Dichloroacetate, an Inhibitor of Pyruvate Dehydrogenase Kinase. Eur. Urol..

[17] Kankotia S., Stacpoole P.W. (2014). Dichloroacetate and cancer: New home for an orphan drug. Biochim. Biophys Acta.

[18] Dunbar E.M., Coats B.S., Shroads A.L., Langaee T., Lew A., Forder J.R., Shuster J.J., Wagner D.A., Stacpoole P.W. (2014). Phase 1 trial of dichloroacetate (DCA) in adults with recurrent malignant brain tumors. Investig. New Drugs.

[19] Chu Q.S., Sangha R., Spratlin J., Vos L.J., Mackey J.R., McEwan A.J., Venner P., Michelakis E.D. (2015). A phase I open-labeled, single-arm, dose-escalation, study of dichloroacetate (DCA) in patients with advanced solid tumors. Investigig. New Drugs.

[20] Schoonjans C.A., Joudiou N., Brusa D., Corbet C., Feron O., Gallez B. (2020). Acidosis-induced metabolic reprogramming in tumor cells enhances the anti-proliferative activity of the PDK inhibitor dichloroacetate. Cancer Lett..

[21] De Preter G., Neveu M.A., Danhier P., Brisson L., Payen V.L., Porporato P.E., Jordan B.F., Sonveaux P., Gallez B. (2016). Inhibition of the pentose phosphate pathway by dichloroacetate unravels a missing link between aerobic glycolysis and cancer cell proliferation. Oncotarget.

[22] Michelakis E.D., Webster L., Mackey J.R. (2008). Dichloroacetate (DCA) as a potential metabolic-targeting therapy for cancer. Br. J. Cancer.

[23] Feron O., Belhassen L., Kobzik L., Smith T.W., Kelly R.A., Michel T. (1996). Endothelial nitric oxide synthase targeting to caveolae. Specific interactions with caveolin isoforms in cardiac myocytes and endothelial cells. J. Biol. Chem..

[24] Bonnet S., Archer S.L., Allalunis-Turner J., Haromy A., Beaulieu C., Thompson R., Lee C.T., Lopaschuk G.D., Puttagunta L., Bonnet S. (2007). A mitochondria-K+ channel axis is suppressed in cancer and its normalization promotes apoptosis and inhibits cancer growth. Cancer Cell.

[25] Bruning U., Morales-Rodriguez F., Kalucka J., Goveia J., Taverna F., Queiroz K.C.S., Dubois C., Cantelmo A.R., Chen R., Loroch S. (2018). Impairment of Angiogenesis by Fatty Acid Synthase Inhibition Involves mTOR Malonylation. Cell Metab..

